# Targeting Hypoglossal Neurobiology in the Treatment of Obstructive Sleep Apnea: The Emergence of Aroxybutynin–Atomoxetine (AD109)

**DOI:** 10.3390/brainsci16080799

**Published:** 2026-07-29

**Authors:** Stanley Wong, Tetyana Kendzerska, Michael S. B. Mak

**Affiliations:** 1 Department of Psychiatry, Temerty Faculty of Medicine, University of Toronto, Toronto, ON M5T 1R8, Canada; 2 Department of Medicine, The Ottawa Hospital, Ottawa, ON K1Y 4E9, Canada; tkendzerska@toh.ca; 3 Faculty of Medicine, University of Ottawa, Ottawa, ON K1H 8M5, Canada; 4 Ottawa Hospital Research Institute, Ottawa, ON K1Y 4E9, Canada; 5 Centre for Addiction and Mental Health, Toronto, ON M6J 1H3, Canada; michael.mak@camh.ca; 6 Department of Medicine, McMaster University, Hamilton, ON L8S 4L8, Canada; 7 Department of Psychiatry, Western University, London, ON N6C 0A7, Canada

**Keywords:** obstructive sleep apnea, AD109, atomoxetine, aroxybutynin, hypoglossal motor nucleus

## Abstract

**Highlights:**

**What are the main findings?**

- Reduced noradrenergic drive and muscarinic inhibition at the hypoglossal motor nucleus suppress pharyngeal dilator tone in sleep, which contributes to obstructive sleep apnea, providing the rationale for AD109 (a combination of the selective noradrenergic reuptake inhibitor atomoxetine and the antimuscarinic aroxybutynin).
- In two phase 3 trials in adults with mild-to-severe obstructive sleep apnea who were intolerant to or declined positive airway pressure, once-daily AD109 produced a model-estimated reduction in the apnea–hypopnea index of roughly 44–47% versus placebo at 26 weeks, with improved nocturnal oxygenation and good safety and tolerability.
- Key side effects for clinicians to consider include dry mouth (dose-dependent and related to the aroxybutynin), insomnia, nausea, and urinary hesitancy. There were no serious treatment-related adverse events reported.

**What are the implications of the main findings?**

- AD109 is a promising oral obstructive sleep apnea (AHI ≥ 5) pharmacotherapy in adults, especially when positive airway pressure is not tolerated or declined.
- Long-term data and comparison with positive airway pressure are needed.

**Abstract:**

Obstructive sleep apnea (OSA) is a highly prevalent sleep-related breathing disorder affecting an estimated 1.36 billion people worldwide. It is associated with excessive daytime sleepiness, impaired quality of life, cardiovascular and cerebrovascular disease, and increased all-cause mortality, and is a substantial economic burden. Although positive airway pressure (PAP) remains the gold-standard therapy, its effectiveness is limited by suboptimal tolerance and adherence, leading to interest in pharmacological alternatives. Among emerging options, AD109, a combination of the selective noradrenergic reuptake inhibitor atomoxetine (75 mg) and the antimuscarinic aroxybutynin (2.5 mg), taken once daily, represents a mechanistically targeted approach that acts on the neuromuscular roots of OSA. Preclinical work demonstrated that pharyngeal dilator tone is suppressed in sleep through withdrawal of noradrenergic excitation and active muscarinic inhibition at the hypoglossal motor nucleus, providing the rationale for combining a noradrenergic agent with an antimuscarinic to augment hypoglossal motor output and preserve upper-airway patency. Building on a series of smaller precursor trials of noradrenergic–antimuscarinic combinations, AD109 has demonstrated consistent efficacy across phase 2 and two large phase 3 trials (SynAIRgy and LunAIRo), with meaningful reductions in the apnea–hypopnea index, improvements in nocturnal oxygenation, and a generally favorable tolerability profile. These findings position AD109 as a promising oral option, particularly for adults with mild to severe OSA who cannot tolerate or decline PAP. However, its effect on long-term cardiovascular, neurocognitive, and mortality outcomes remains to be established. This commentary reviews the mechanism of action and clinical trial evidence for AD109 and related combinations.

## 1. Introduction

Obstructive sleep apnea (OSA) is a common sleep-related breathing disorder that affects approximately 1.36 billion people globally as of 2019 (936 million with mild to moderate severity and 425 million with moderate to severe severity) [1]. OSA is commonly comorbid with obesity, hypertension, type 2 diabetes, atrial fibrillation, and depression, relationships that are often bidirectional [2]. Risk factors include increasing age, higher BMI, and male sex [3]. Symptomatically, OSA manifests as excessive daytime sleepiness, low mood or concentration difficulties. However, it can also appear asymptomatically and can also be associated with serious health consequences, including cardiovascular disease, cerebrovascular disease, effects on cognition, and increased risk of all-cause mortality [4,5,6]. Furthermore, it is estimated to have an economic effect in the billions, as excessive daytime sleepiness is associated with indirect economic effects, such as low mood, decreased activity, decreased quality of life, accident-related near-misses, and motor vehicle collisions [7,8]. In the United States, the diagnosis and treatment of OSA cost the healthcare system approximately $12.4 billion USD in 2015 [9].

Given the significant healthcare and economic effects of OSA, treatment of it is necessary. The gold-standard treatment options for OSA include positive airway pressure (PAP) therapy [10]. However, PAP is not always well tolerated due to various reasons, including costs, claustrophobia, and patient preference [11,12,13]. Furthermore, non-adherence to continuous PAP (CPAP) is substantial, as it is estimated that 34% of users are not adherent with treatment [14]. Other treatments include oral appliance therapy, lifestyle changes (e.g., weight loss or positional therapy), hypoglossal nerve stimulation, or surgeries (e.g., bariatric surgery or airway surgery for abnormal airways) [15,16,17].

There is also an emerging role for medication treatments to treat OSA, as medications may potentially be more acceptable or tolerable compared with wearing a CPAP device and are less invasive than surgical interventions. Medications such as glucagon-like peptide 1 (GLP-1) receptor agonists induce weight loss, in which obesity is a significant contributor to OSA [18,19]. Tirzepatide is the first GLP-1 receptor agonist approved by the US Food and Drug Administration (FDA) for the treatment of adult patients with moderate to severe OSA that is complicated by obesity, making it the first approved medication for direct OSA treatment [20]. However, it is only indicated in people with a BMI of 30 kg/m^2^ or greater and requires weekly injections. Furthermore, GLP-1 receptor agonists have side effects such as nausea and vomiting and other gastrointestinal side effects, which may not be as well tolerated [21].

Another emerging medication is a combination of selective noradrenergic reuptake inhibitor (SNRI) atomoxetine and the anti-muscarinic drug aroxybutynin. In particular, AD109 is a specific formulation of 75 mg of atomoxetine and 2.5 mg of aroxybutynin created by Apnimed [22]. Apnimed has submitted a new drug application to the FDA, but the application has not yet been reported as accepted for review [23]. AD109 and similar combination medications work by targeting the neuromuscular roots of OSA by stimulating the hypoglossal motor nucleus (HMN). This could be a novel pharmacotherapy that targets the neuromuscular and neurological causes of OSA. This commentary will explore the mechanism of action of AD109 and similar medications and the clinical trial evidence.

In addition to summarizing the current evidence, we aim to advance the view that AD109 signals a broader shift in how OSA may be conceptualized and treated. For decades, management has centered on mechanically splinting a vulnerable airway. However, AD109 represents the first pharmacological intervention targeting the neuromuscular contribution to airway collapse that has received regulatory approval. Within the current existing treatments for OSA, we believe that AD109’s role will not be to replace PAP therapy but rather to expand effective treatment to the large proportion of patients who decline or have challenges with PAP therapy and who go untreated.

## 2. Mechanism of Action of Atomoxetine and Aroxybutynin

Obstructive sleep apnea is a heterogeneous condition arising from a combination of anatomical and non-anatomical contributors. It can be described by four key pathophysiological traits, which include a highly collapsible airway (increased upper-airway collapsibility), impaired upper-airway dilator muscle responsiveness, unstable ventilatory control (high loop gain), and a low respiratory arousal threshold [24]. The relative contributions of these traits vary between individuals, but this framework has been used to identify rational targets for treatment. AD109 and similar combination medications of noradrenergic and anti-muscarinic agents work on impaired dilator muscle responsiveness and on upper-airway collapsibility by augmenting HMN output during sleep with lesser effects on loop gain and little effect on the arousal threshold.

In OSA, there is often anatomical vulnerability in the individual through a narrow or highly collapsible pharynx as well as a sleep-dependent loss of compensatory neuromuscular tone [25]. While awake, people with this anatomical vulnerability in their airways compensate through increased activity of the genioglossus and other pharyngeal dilator muscles. However, at the onset of sleep, this compensatory activity fails, which leads to the pharynx consequently becoming susceptible to collapse [25].

The preclinical evidence has clarified the mechanisms underlying this sleep-dependent failure of compensatory tone at the level of the hypoglossal motor nucleus (HMN). Work in animal models suggests that two processes converge to suppress pharyngeal dilator activity in sleep. The first is disfacilitation, in which excitatory drives are most consistent [26]. Noradrenergic inputs acting on the alpha-1 receptors are progressively withdrawn from wakefulness to NREM to REM sleep [27,28]. The second is active inhibition, mediated by a muscarinic receptor pathway linked to G-protein-coupled potassium channels, whose inhibitory influence is greatest in REM sleep [29,30,31]. These findings suggest that restoring noradrenergic excitation while blocking muscarinic inhibition could offset the neuromuscular loss that leads the pharynx to become more susceptible to collapse [25]. This also provided the rationale for the combination of atomoxetine with aroxybutynin. Atomoxetine, which is an SNRI, would theoretically increase synaptic noradrenaline and enhance central excitation to the upper airway motor neurons, which would improve the muscle tone of the dilator muscles, while aroxybutynin, an anti-muscarinic medication, has been theorized to reduce muscarinic inhibition of the HMN activity.

## 3. Precursor SNRI and Anti-Muscarinic Combination Studies

Prior to AD109, previous trials have investigated the combination of an SNRI and anti-muscarinic medication for the treatment of OSA, which laid the foundation for the development of AD109.

The seminal study by Taranto-Montemurro et al. randomized 20 patients to a single night of the combination of 80 mg of atomoxetine and 5 mg of oxybutynin vs. placebo and found a marked reduction in the apnea–hypopnea index (AHI) from 28.5 to 7.5 events per hour, which was a 63% reduction that was statistically significant [32]. There was also a three-fold increase in genioglossus responsiveness. Interestingly, when each agent was given alone, the AHI was not found to be reduced, which further supports the combinatorial medication approach. The authors also conducted a companion endotyping analysis of the same cohort and attributed the improvements in their AHI to the reduced pharyngeal collapsibility and improved muscle compensation [33]. However, these findings must be interpreted with caution as the data were derived from a single dose in a small sample that provided no data on any residual effects.

Aishah et al. conducted a double-blind randomized crossover trial of 10 individuals with severe OSA who were given either a combination of 80 mg of atomoxetine with 5 mg of solifenacin succinate or 2 mg of biperiden hydrochloride or a placebo [34]. It was found that there were only modest changes in AHI relative to the placebo, but it was not statistically significant. However, the reductions in the AHI compared with reductions in the AHI that were previously reported in studies with oxybutynin were modest in comparison, which implied that broader muscarinic receptor selectivity, specifically M2 affinity, may be important for pharyngeal muscle activation.

Lim et al. investigated the combination of 4 mg of reboxetine combined with 20 mg of hyoscine butylbromide in 12 patients [35]. The AHI was decreased by approximately 35% while improving tensor palatini activity and upper-airway function during NREM sleep. However, it was also found that there was altered sleep architecture in the form of increased N2 and suppressed REM, reduced sleep efficiency, and anti-muscarinic side effects, including tachycardia, dry mouth, and urinary hesitancy. Messineo et al. investigated the combination of 80 mg of atomoxetine with 4 mg of fesoterodine in 12 patients for a single night and found that it did not reduce the overall AHI, although there were improvements in oxygen saturation [36].

Schweitzer et al. conducted a larger phase two trial of AD036, which was the combination of 80 mg of atomoxetine with 5 mg of oxybutynin [37]. Sixty-two participants were randomized to AD036, atomoxetine alone, or placebo. Both active arms reduced the AHI (AD036 = 6.2 events/hour and atomoxetine alone = 4.8 events/hour), with improved airway collapsibility. However, only AD036 improved ventilation at the arousal threshold, which was consistent with a stronger effect on the upper-airway dilator muscles.

**Figure 1:**
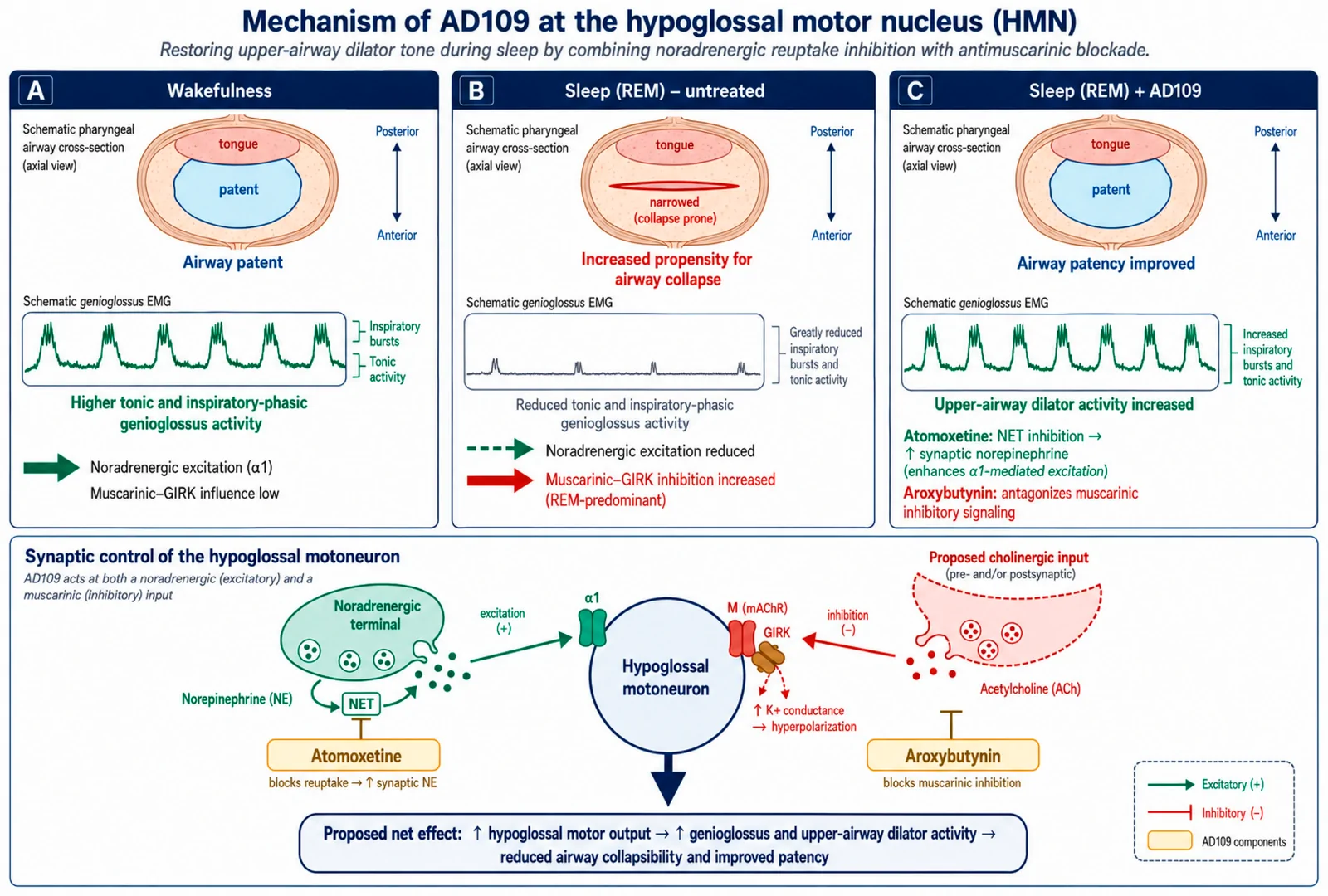
Proposed mechanism of AD109 at the hypoglossal motor nucleus (HMN). During REM sleep, reduced noradrenergic excitation and increased muscarinic–GIRK inhibition decrease genioglossus activity and promote airway collapse. Atomoxetine increases synaptic noradrenaline, while aroxybutynin blocks muscarinic inhibition, which increases hypoglossal motor output and improves airway patency.

When viewed together, these precursor trials do more than establish proof of concept. They reveal why treatment responses were varied and how the optimal combination was determined. It was the consistent finding that atomoxetine paired with the non-selective antimuscarinic oxybutynin produced larger reductions in the AHI than combinations using the more receptor-selective agents, such as solifenacin, biperiden, or fesoterodine. The broad muscarinic blockade and M2-receptor affinity in oxybutynin was important for restoring pharyngeal dilator activity. The choice of noradrenergic agent also mattered as well. Reboxetine, which has a longer half-life than atomoxetine, produced comparable benefit but at the cost of altered sleep architecture. This finding highlighted the tension between motor reactivation and sleep continuity, which led to the choice of atomoxetine. Additionally, response was further shaped by phenotype, with the greatest benefit in patients whose airways were less severely collapsible. These observations converged and led to the pairing of a noradrenergic reuptake inhibitor with a broadly active antimuscarinic, while mitigating the tolerability burden of racemic oxybutynin. Aroxybutynin, the R-enantiomer that carries the majority of oxybutynin’s antimuscarinic activity while reducing exposure to the less favorable S-enantiomer, was, therefore, selected and combined with atomoxetine to create AD109. Figure 1 visualizes the proposed mechanism of AD109 at the HMN.

Overall, these precursor studies provided a consistent proof of concept that the combination of an SNRI with an anti-muscarinic could augment pharyngeal dilator activity and improve airway patency during sleep. However, most of these studies were limited by small sample sizes, brief periods of intervention, and lack of long-term efficacy and safety data.

## 4. Aroxybutynin–Atomoxetine Combination (AD109)

AD109 is the combination of 75 mg of atomoxetine and 2.5 mg of aroxybutynin. Aroxybutynin is the R-enantiomer of oxybutynin with the most anti-muscarinic activity [25,38]. It is taken as a once-daily oral fixed-dose combination. We have summarized the trials of AD109 in Table 1.

Rosenberg et al. conducted an initial randomized double-blind placebo-controlled crossover study in adults with mild to moderate OSA with AD109 in a 75 mg and 2.5 mg combination and a lower 37.5 mg and 2.5 mg combination compared with placebo across three overnight periods in 32 participants [38]. It was found that AD109 reduced OSA severity from a median of 13.2 (IQR: 8.0–19.1) events/hour on placebo to 5.5 (2.2–9.6) events/hour on the high dose and to 7.8 (4–13.7) events/hour on the lower dose. From a safety perspective, AD109 demonstrated a favorable safety profile. Schweitzer et al. then conducted the phase 2b MARIPOSA trial, which was a one-month randomized double-blind placebo-controlled trial of 211 adults with mild to severe OSA across 25 sites in the United States [22]. Participants were randomized to one of four arms, which were AD109 in a 75 mg and 2.5 mg combination, a 75 mg and 5 mg combination, 75 mg of atomoxetine alone, and a placebo. Both AD109 arms achieved a significant reduction in the AHI relative to placebo, where approximately 43–44% achieved at least a 50% AHI reduction, versus 16.6% with the placebo (median [IQR]: of 20.5 [12.3–27.2] to 10.8 [5.6–18.5] in the 75  mg and 2.5 mg of AD109 arm; 19.4 [13.7–26.4] to 9.5 [6.1–19.3] in the 75 mg and 5 mg arm; and 19.0 [11.8–28.8] to 11.8 [5.5–21.5] with atomoxetine alone). The increased aroxybutynin dosage from 2.5 to 5 mg did not improve efficacy but increased antimuscarinic adverse effects, which supports the selection of 75 mg and 2.5 mg. Common side effects reported were dry mouth, insomnia, urinary hesitation, and nausea.

Two large phase 3 trials subsequently evaluated the fixed-dose combination selected from MARIPOSA (75 mg of atomoxetine with 2.5 mg of aroxybutynin once daily at bedtime). Strollo et al. conducted the SynAIRgy trial [39]. This was a 26-week randomized double-blind placebo-controlled trial of 646 adults with mild to severe OSA who had failed or declined PAP therapy. This study was conducted across 69 centers in the United States and Canada. AD109 reduced the AHI by approximately 44% relative to placebo at week 26 (the AHI treatment difference was −4.0 events/hour [95% CI: −6.4 to −1.6]), with 51.2% of treated participants experiencing a reduction in the disease-severity category and 22.3% achieving complete disease control. There was also significant improvement in oxygenation, as measured by hypoxic burden and the oxygen desaturation index. Patel et al. conducted the companion LunAIRo trial, which was a 51-week parallel-arm study with its primary endpoint assessed at 26 weeks in 660 adults [40]. This study was conducted across 64 centers in the United States. Similarly, they reported a mean AHI reduction of 46.8% from baseline at week 26 vs. 6.8% with placebo (*p* < 0.001), and approximately 38.3% of participants achieved a reduction in the AHI of ≥50%. This remained significant at week 51. They also found a reduction in hypoxic burden and the oxygen desaturation index. Across both trials of AD109, it was generally well tolerated, with adverse events that were more frequent than with placebo (71.4% vs. 47.3% in SynAIRgy) [39]. Most of the adverse events were mild to moderate in nature and consistent with the drug’s anti-muscarinic and noradrenergic actions, including dry mouth, insomnia, nausea, urinary hesitancy, constipation, and somnolence [39,40]. There were no serious treatment-related adverse events reported [39,40]. Despite the strengths of these findings, it should be noted that the residual on-treatment AHI and the proportion of patients achieving complete disease control indicated that AD109 reduces but does not resolve OSA in most patients.

In order to address the long-term safety of chronic dosing beyond the clinical trial periods, an ongoing Phase 3 open-label continuation study (NCT06566820) is enrolling participants who completed either SynAIRgy or LunAIRo and treats them with open-label AD109, with the aim of characterizing the long-term safety, tolerability, and maintenance of efficacy of the combination [41]. Such extension data will be important to establish whether the reductions in OSA severity and the improvements in nocturnal oxygenation observed are sustained with continued use and, thus, to clarify the role of AD109 in the chronic pharmacological management of OSA.

In the landscape of OSA treatments, AD109 is best understood not as a replacement for existing therapies but as an additional option occupying a distinct niche. CPAP/PAP remains the most efficacious treatment and can normalize the AHI in adherent users, but real-world adherence is limited. Oral appliances offer an appliance-based alternative with their own tolerability, cost, and eligibility constraints. Hypoglossal nerve stimulation additionally requires surgical implantation and specific anatomical criteria. Weight loss pharmacotherapy, exemplified by tirzepatide and GLP-1 receptor agonists, addresses the obesity that drives airway collapse but is restricted to patients with obesity or diabetes. They also require regular injections and carry gastrointestinal side effects. AD109 offers a once-daily oral therapy that is non-invasive, mechanism-based, and applicable across a broad severity and weight range. However, it reduces rather than abolishes OSA and lacks long-term outcome data. Its most rational current role is as an oral option for adults who cannot tolerate or decline PAP and, potentially, as a component of combination strategies, e.g., alongside weight loss therapy, that simultaneously address anatomical and neuromuscular contributors.

## 5. Future Considerations

The early phase 2 and 3 trials show productive directions for the continued development of AD109. The benefits are statistically robust, and there were consistent improvements in oxygenation and hypoxic burden. Given the cardiovascular, neurocognitive, and mortality burdens of OSA, it would be important for future studies to assess any potential reduction in these aspects. Similarly, given the variation in the subjective responses of improvements in fatigue, it would be beneficial to have future studies identify participants that would most benefit from AD109. Attention to tolerability will also be important, given that roughly 21% of participants discontinued AD109 for adverse events in SynAIRgy, suggesting that real-world adherence, dose optimization, and side-effect management require further study. Taken together, these considerations can further reinforce the potential significance of AD109 in the treatment of OSA.

Another important direction for future work is also to identify traits that best predict response to AD109, which would enable a precision medicine approach to patient selection. Gell et al., using gold-standard intraesophageal catheter physiology in 58 participants, found that the arousal threshold was the only statistically significant modifier of response, with high-arousal-threshold patients deriving substantial benefit and low-threshold patients deriving essentially none [42]. Furthermore, non-significant but consistent trends favored lower baseline muscle effectiveness and milder collapsibility, whereas loop gain did not modify response [42]. These findings align with earlier endotyping work that showed that patients with a lower AHI, higher passive airway patency, and a higher proportion of hypopneas were most likely to achieve a complete response [33]. Therefore, the ideal candidate might appear to have a higher arousal threshold, milder collapsibility, and lower dilator muscle effectiveness. However, no clinically ready non-invasive biomarker yet exists to identify such responders, and prospective validation remains a priority.

Additionally, residual disease is an important consideration. Although AD109 lowered the AHI substantially in relative terms, some patients retained clinically relevant OSA, and only about one-fifth achieved complete disease control. It is currently unknown whether this partial reduction in AHI confers any cardiovascular or neurocognitive protection that has been historically attributed to full, CPAP-mediated control. Future studies should prioritize these cardiac and neurocognitive outcomes for long-term health.

Safety with chronic use also merits long-term investigation. It was observed that AD109 approximately halved the median REM sleep over six months, which is a non-trivial finding given the role of REM sleep in memory consolidation and emotional regulation, and its long-term neuropsychiatric and cognitive consequences are unknown. Similarly, although aroxybutynin was designed as the R-enantiomer to preserve peripheral antimuscarinic efficacy while limiting central exposure, there is a cumulative anticholinergic burden of chronic therapy, particularly in older adults. Increased anticholinergic exposure has been associated with cognitive decline and warrants ongoing monitoring. These considerations are being addressed by the open-label continuation study, which will provide valuable safety data, but adequately powered, long-term, outcome-oriented trials will still be needed.

## 6. Conclusions

There is emerging evidence for the combination of a noradrenergic agent and an anti-muscarinic for the treatment of OSA, more specifically AD109, a once-daily oral fixed-dose combination of atomoxetine and aroxybutynin. It targets the neuromuscular roots of OSA by augmenting hypoglossal motor output, which offsets the sleep-dependent loss of pharyngeal dilator tone, which predisposes the airway to collapse. AD109 has now demonstrated consistent efficacy across phase 2 and two large phase 3 trials, which have shown meaningful reductions in the AHI, with substantial improvements in nocturnal oxygenation as well as a generally safe profile. These trials show it as a promising oral medication option, particularly for patients who cannot tolerate or decline PAP therapy. Its benefits relative to PAP as well as long-term effects on cardiovascular, neurocognitive, and mortality-related outcomes remain to be established and are an area for potential further investigation. Long-term efficacy and safety data are also warranted and are currently underway. Overall, AD109 represents a neurobiological and mechanistically targeted pharmacotherapy for OSA, and further studies will be essential to clarify its role in the management of this common and important disorder.

## Figures and Tables

**Table 1:** Clinical trials of AD109 for obstructive sleep apnea.

Trial/Author	Phase/Design/*N*	Duration	Demographics and BMI	Baseline AHI and Severity	Primary Efficacy Findings	Key Secondary Findings	Major Adverse Events
Rosenberg et al. [38]	Phase 1b; RCT, double-blind, crossover, 3-period; *N* not specified	3 single nights (crossover)	Adults; 35% female; median age: 53 years (IQR: 47–62), and median BMI: 30.4 kg/m^2^ (27.0–33.1)	Median AHI: 13.2 (IQR: 8.0–19.1); mild to moderate	AHI decreased from 13.2 to 5.5 (high dose: 75/2.5 mg; *p* < 0.001) AHI decreased from 13.2 to 7.8 (low dose: 37.5/2.5 mg; *p* < 0.05)	Hypoxic burden decreased from 13.9 to 2.3 (%min)/h (*p* < 0.001); ODI significantly reduced	Dry mouth
Schweitzer et al. [22] [MARIPOSA]	Phase 2; RCT, double-blind, parallel-group, 4-arm; *N* = 211 (181 analyzed)	4 weeks	Adults; 41% female, median age: 55 years (IQR: 48–60), and median BMI: 32.2 kg/m^2^ (IQR: 28.0–35.2)	AHI_4_%: 19.0–20.5 across arms; mild to moderate predominant	AHI_4_% decreased by 47.1% (AD109: 2.5/75; *p* < 0.0001) AHI decreased by 42.9% (5/75; *p* < 0.0001); AHI decreased by 38.8% (atomoxetine alone; *p* < 0.01)	Fatigue improved with AD109 2.5/75 (*p* < 0.05 vs. placebo); atomoxetine alone decreased TST (*p* < 0.05)	Dry mouth, insomnia, urinary hesitancy
Strollo et al. [39] [SynAIRgy]	Phase 3; RCT, double-blind, parallel-group; *N* = 646	26 weeks	Median: 58 yr; 49.3% female; median BMI: 32.4	Median AHI: 19.6; 35% mild, 42% moderate, 23% severe	AHI treatment difference was −4.0 events/h (95% CI: −6.4 to −1.6; *p* = 0.001) Model-estimated decrease of 44.1% vs. 17.6% (*p* < 0.0001)	ODI and hypoxic burden improved; PROMIS-Fatigue: no significant difference	Dry mouth, nausea, insomnia, urinary hesitation (no serious treatment-related AEs; 21.2% vs. 3.1% discontinuation)
Patel et al. [40] [LunAIRo]	Phase 3; RCT, double-blind, parallel-group; *N* = 660	51 weeks	Adults; 46% female	37% mild, 33% moderate, 30% severe	Model-estimated AHI_4_ reduction of 46.8% at wk. 26 vs. 6.8% with placebo (*p* < 0.001)	Hypoxic burden of −58.2% and ODI improved (*p* < 0.0001)	Dry mouth, insomnia, nausea, urinary hesitancy, somnolence, headache

Abbreviations: AHI, apnea–hypopnea index; AHI_4_%, AHI scored using a ≥4% oxygen desaturation criterion. Studies included [22,38,39,40].

## Data Availability

No new data were created or analyzed in this study. Data sharing is not applicable to this article.

## Abbreviations

The following abbreviations are used in this manuscript:

AHI	Apnea–hypopnea index
BMI	Body mass index
CI	Confidence interval
CPAP	Continuous positive airway pressure
FDA	US Food and Drug Administration
GLP-1	Glucagon-like peptide-1
HMN	Hypoglossal motor nucleus
IQR	Interquartile range
NREM	Non-rapid eye movement
ODI	Oxygen desaturation index
OSA	Obstructive sleep apnea
PAP	Positive airway pressure
REM	Rapid eye movement
SNRI	Selective noradrenergic reuptake inhibitor
